# Radiographic film dosimetry for IMRT fields in the near‐surface buildup region

**DOI:** 10.1120/jacmp.v9i4.2782

**Published:** 2008-10-24

**Authors:** Peter L. Roberson, Jean M. Moran, Ravi Kulasekere

**Affiliations:** ^1^ Department of Radiation Oncology University of Michigan Medical Center Ann Arbor Michigan U.S.A.

**Keywords:** IMRT, buildup dose, radiographic film, quality assurance

## Abstract

Radiographic film dosimetry provides fast, convenient 2‐D dose distributions, but is challenged by the dependence of film response on scatter conditions (i.e., energy dependence). Verification of delivered dose in the surface buildup region is important for intensity modulated radiation therapy (IMRT) when volumes of interest encroach on these regions (e.g., head/neck, breast). The current work demonstrates that film dosimetry can accurately predict the dose in the buildup region for IMRT, since 1) film dosimetry can be performed with sufficient accuracy for small fields and 2) IMRT is delivered by a series of “small” segments (step and shoot IMRT). This work evaluates the accuracy of X‐OMAT V (XV) and Extended Dose Range (EDR) film for measurements from 2 mm to 15 mm depths for small fields and clinical IMRT beams. Film measurements have been compared to single point measurements made with a stereotactic diode and parallel plate ionization chamber (P11) and thermoluminescent dosimeters (TLD) at various depths for square (diode, P11) and IMRT (diode, TLD) fields. Film calibration was performed using an 8‐field step exposure on a single film at 5 cm depth, which has been corrected to represent either small field or large field depth dependent film calibration techniques. Up to 10% correction for film response variation as a function of depth was required for measurements in the buildup region. A depth‐dependent calibration can sufficiently improve the accuracy for IMRT calculation verification (i.e., ≤5% uncertainty). A small field film calibration technique was most appropriate for IMRT field measurements. Improved buildup region dose measurements for clinical IMRT fields promotes improved dose estimation performance for (inverse) treatment planning and allows more quantitative treatment delivery validation.

PACS numbers: 87.53.‐j, 87.53.Dq

## I. INTRODUCTION

Accurate dose measurement in the near‐surface buildup region is necessary for the validation of dose calculation algorithms used for treatment planning. It is particularly important for treatment sites, such as head and neck, breast or chest wall, that contain normal tissue or target volumes in the surface regions (less than the depth of maximum dose) and where dose calculation algorithms are being used to optimize dose distributions via inverse planning techniques.^(1)^ Step‐and‐shoot intensity modulated radiation therapy (IMRT) fields resulting from inverse planning are essentially a collection of small fields grouped to yield a larger modulated field. Since the surface dose is strongly dependent on collimator settings, it is also expected to be dependent on the treatment delivery parameters (e.g., sequencing design) for IMRT fields.

The switch of head and neck treatment from conformal fields to IMRT has resulted in an increase in skin toxicity.^(2)^ This increase was attributed to increased near‐surface dose due to the use of improved immobilization techniques (masks) combined with the use of multiple field IMRT techniques, effectively increasing oblique incidence on the skin. However, it has been reported that the IMRT fields themselves reduce near‐surface dose compared to conformal fields.^(3)^

Treatment planning system dose calculations have historically been less accurate in near‐surface buildup regions. Reasons for this include the difficulty of dose calculation of accelerator‐setup‐dependent contamination radiation, the effect of oblique incidence on the buildup dose, and the increased challenge of measuring dose contamination and buildup dose. Task Group Report 53 ^(4)^ on quality assurance for clinical radiotherapy treatment planning systems recommends performance criteria in the buildup region that are much more forgiving compared to in‐field regions at depths of $d_{\text{max}}$ and greater (20% to 50% in buildup region compared to 1% to 5% in‐field regions). It has been reported that some commercial treatment planning systems over estimated the near‐surface buildup region dose by 7 to 19%.^(5)^

Strategies for targeting near‐surface volumes include:^(1)^ 1) identifying a skin volume as an organ‐at‐risk; 2) modifying the planning target volume to avoid the skin organ‐at–risk; 3) creating a bolus structure solely for planning purposes to minimize the effect of using buildup dose to improve target coverage; 4) removing beamlets in the near‐surface regions post optimization. It is important to have an accurate estimate of near‐surface dose even if it is machine and setup dependent.^(6)^

Radiographic film dosimetry provides a fast, convenient method of measuring 2D dose distributions in megavoltage radiation beams. However, disadvantages include the energy dependence of film response and processor variation induced errors. Many recent studies have been performed to investigate the use of film for IMRT dose evaluation at depths of maximum dose or greater^(^ ^7^ ^–^ ^10^ ^)^ and in the surface buildup regions.^(11)^ The present study examines the validity of using film to measure dose in the near‐surface buildup region in clinical IMRT fields. Specifically, we have estimated the errors resulting from the use of film for near‐surface buildup region dosimetry, using a depth and field size dependent calibration scheme, which was subsequently corrected for processor response variations. A field size of 3×3 cm was chosen to represent a small field calibration to account for the effects of the piecewise construction of the IMRT fields, and a field size of 20×20 cm was chosen to represent the large field (jaw aperture) calibration to account for the effects of accumulated scatter at low dose levels in the IMRT field. The dose measured using film in the buildup region was compared to parallel plate ionization chamber (Attix and P11 chambers), stereotactic diode and thermoluminescent dosimeter (TLD) measurements. The stereotactic diode was chosen due to its small detector volume ^(12)^ ^,^ ^(13)^ and relative accuracy of surface measurements.^(3)^ The technique was tested on a sampling of clinical IMRT fields. An accuracy goal of 5% or less was achieved in the buildup region for both Kodak X‐Omat V (XV) and Kodak Extended Dose Range (EDR) film using an appropriate film calibration scheme.

## II. MATERIALS AND METHODS

All measurements were performed on a Varian Clinac 21‐EX accelerator (Varian Medical Systems, Palo Alto, CA) using a 6MV photon beam and a Varian Millennium 120 multi‐leaf collimator (MLC). Films used in the experiments were Kodak Ready‐Pak type X‐Omat V (XV), XV2, and Extended Dose Range (EDR), EDR2, film. EDR film was given twice the exposure (two beam‐on exposures for IMRT fields) relative to XV film to better match its response range. Films for each experiment were processed over a reasonable period of time on a Kodak X‐Omat 3000RA automatic processor. The processor was subjected to a rigorous quality control test before experiment films were processed. Films were digitized on a Lumiscan LS‐75 (Lumisys, Sunnyvale. CA) laser digitizer and data were analyzed using the University of Michigan treatment planning software (UMPLAN). Film response was converted to optical density using a standard step‐wedge provided by Kodak (Eastman Kodak Co., Rochester, NY). Gammex RMI Model 457 (Gammex RMI, Middleton, WI) 30×30 cm Solid Water slabs (in perpendicular geometry) were used as the phantom for all film measurements. A Med‐Tec (CIVCO Medical Solutions, Orange City, IA) water phantom and a Scanditronix (Scanditronix‐Wellhofer North America, Bartlett, TN) stereotactic diode (0.06 mm thickness and 0.6 mm diameter sensitive volume) were used to perform diode measurements on square and IMRT fields. A waterproof Exradin P11 parallel plate chamber (Standard Imaging, Middleton, WI) with a 2 mm gap, 1 mm polystyrene window, 200 mm collecting diameter and 7 mm guard ring was used to determine the depth dose of the large (20×20 cm) field size. The Rawlinson^(14)^ correction factor was used to correct the parallel plate measurements.

Depth dose values for the small (3×3 cm) field size in the buildup region were obtained using LiF TLD. The small (3.18×3.18 mm) size of the TLD is ideal for point dose small field measurements. Since the thickness of the TLD can bias the results in the buildup region, an extrapolation method^(15)^ using TLD chips of 0.89 mm, 0.38 mm and 0.15 mm was used to extrapolate to dose at zero thickness for measurements shallower than depth of maximum dose. The TLD chips were calibrated at a 10 cm depth using a 10×10 cm field size to determine individual sensitivity factors. A Solid Water TLD holder with custom machined cavities of different thicknesses was used for measurements. The TLD chips were handled using a vacuum device to prevent contamination. Prior to measurements the TLD chips were annealed for 1 hour at 400 °C and 2 hours at 100 °C. After irradiation the TLD chips underwent a preread heat cycle of 10 minutes at 100 °C and were read out using a Harshaw reader (Thermo Scientific, Waltham, MA). The average of three measurements for each thickness of TLD was used to extrapolate to zero thickness.

Depth dose measurements were also performed with TLD and diode using a 5×5 cm field size and were compared with Attix (1 mm gap, 12.7 mm diameter collector, 13.5 mm guard ring, $4.8\;\text{mg}/{\text{cm}}^{2}$ entrance window, used in Solid Water) and Pll parallel plate chamber (in water). These measurements were used to help validate the near‐surface response of each experimental measurement technique.

Experimental film exposures were performed at 90 SSD in Solid Water at 4 depths (2 mm, 5 mm, 15 mm, and 100 mm). Film response was calibrated at each depth and field size (3×3 cm, 20×20 cm) of interest. For each depth, the film calibration curve consisted of 8 exposure levels each exposed at central axis on individual films, at dose levels closely matched to levels for a reference calibration film taken at 95 SSD and 5 cm depth. A reference film was exposed at the same time as the calibration curve exposures. A quadratic polynomial was fit to the dose response data. Individual experiment runs were related to the calibration curves using calibration exposures.

The calibration films were composed of eight 3×3 cm fields exposed to escalating dose levels using jaws and MLC to minimize scatter and transmission dose.^(16)^ ^,^ ^(17)^ Dose at each of the 8 exposure positions was measured using an Exradin A14 ion chamber, including effects of primary beam, collimator transmission and in‐phantom scatter.

The correction for film processing variation was achieved by establishing a reference 8‐step calibration film and by correcting each subsequent 8‐step calibration film response to the response of the reference film as outlined below. The difference between the response of an 8‐step calibration film taken during any experiment and that of the reference film is given by

(1) $$C(D)=[(T_{R}(D)-F_{R})-(T_{E}(D)-F_{E})]/(T_{R}(D)-F_{R})$$

where $F_{E}$ and $F_{R}$ are the film fog level of the experiment and reference films, $T_{R}$(D) is the response of the 8‐step reference calibration film, $T_{E}$(D) is the response of the 8‐step experiment calibration film and C(D) is the difference between the reference and experiment calibration film response (with respect to the fog levels) at dose D. The experimental readings are corrected to match the reference readings,

(2) $$\left[T_{C}(D)-F_{C}]=[T_{R}(D)-F_{R}\right]$$

where $T_{C}$ is the experiment calibration film response corrected for processing variations. Therefore from Equations (1) and (2), and assuming a constant fog level $\left(F_{R}=F_{c}=F\right)$,

(3) $$T_{C}(D)=(T_{E}(D)-C(D)*F)/(1-C(D)).$$

The difference data, C(D), was fit to a linear function of dose for each processor run and incorporated into Equation (3) to provide the correction for processor variation. $T_{C}$(D) is the corrected calibration curve applied to the experiment films. This procedure is similar to a single or double‐hit theory of film exposure,^(18)^ ^,^ ^(19)^ provided the exponential in the theoretical response function is expanded in powers of film sensitivity with the calibration curve truncated at the second power and the (relatively small) correction function truncated at the first power. However, the correspondence to the theory is not rigorous since all parameters are phenomenologically determined.

IMRT field measurements were performed in water using three pre‐selected clinical IMRT beams and the diode. Leakage for the diode was tracked at intervals and data were corrected as necessary. The diode measurements were performed at the center of each 1×1 cm beamlet across the beam profiles at close proximity to the central axis position. Response corrected film calibrations were compared to the diode measured dose values for the IMRT fields. Residual errors were calculated for both EDR and XV film for evaluating the accuracy of dose measurement in the buildup region for IMRT fields.

### A. Error Analysis

Film response errors include processor variations and an energy dependent non‐linear response. Corrections were applied for response deviations due to the energy dependence, including response changes due to field size and depth. Processing variation errors were minimized by using a reference 8‐step calibration film exposed and processed with each experiment. The response relationship between the single film 8‐step calibration and multi‐film central axis (field size and depth dependent) calibrations were determined.

Diode measurement error estimates accounted for error in depth setup in a dose gradient region, leakage correction, reproducibility of response and drift in calibrated response. The in‐field energy response dependence was negligible.^(3)^ Measurement error was estimated to be 2% or less (one standard deviation), with the error in depth setup contribution dominant.

TLD extrapolation method measurement error was determined from variations in readings and propagation of error. The extrapolation method used three times as many readings as the non‐extrapolation method, but resulted in comparable measurement uncertainty due to contributions from propagation of error. One standard deviation error was estimated to be 2.5%.

## III. RESULTS

Measurements using the extrapolation TLD method in Solid Water were consistent with parallel plate and diode measurements in water within the estimated measurement error (Table 1). We have noted a systematic difference in the dose to Solid Water and water at 2 mm depth^(3)^ of up to 5%, and have therefore listed comparisons in water and Solid Water separately in Table 1. Buildup dose measurements for larger fields comparing diode and parallel plate techniques in water agreed within 2%. Percentage depth dose results for 3×3 cm are higher than for 5×5 cm because of the 10 cm depth normalization. Percent differences are relative to the normalization depth.

**Table 1:** TLD measurements in Solid Water at 3×3 cm and at 5×5 cm compared to parallel plate measurements in Solid Water (Attix) and diode measurements in water compared to parallel plate measurements in water (P11) as a function of depth. Data were normalized to 10 cm depth (90 cm SSD). Percent differences are relative to the normalization depth.

	3×3 cm			5×5 cm			
*Depth*	*TLD in SW*	*TLD in SW*	*ATTIX in SW*	*% Difference*	*diode in water*	*P11 in water*	*% Difference*
2 mm	98.9	92.0	94.1	−2.2	95.6	98.9	−3.3
5 mm	139.9	133.7	136.5	−2.1	137.1	139.3	−1.6
15 mm	172.8	163.3	163.1	−0.2	162.3	164.4	−1.3

**Figure 1:**
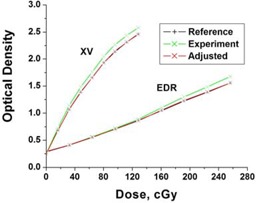
Application of the correction function to the (experiment) calibration film (XV and EDR) to account for film processor variations.

Film response curves vary depending on processor conditions. As illustrated in Fig. 1, film response for a particular experiment varies from a pre‐defined reference response. The experiment response can be corrected to represent the reference response, shown using the 8‐step calibration film technique. By applying the correction function outlined in Equations 1 to 3, the calibration film response from another experiment can be adjusted to the reference response with a standard error of about ±1.5%. Response differences were measured between the 8‐step calibration and the central axis calibration curves for the small and large fields as a function of depth. Corrections were used in the small field and large field film calibrations at each depth in order to analyze the dose distributions for IMRT beams.

**Figure 2:**
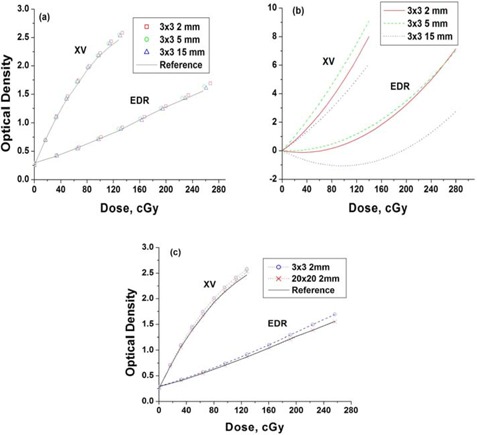
Radiographic film response at depth vs reference calibration response. (a) 3×3 cm central axis film (XV and EDR) calibrations (90 cm SSD) at various depths compared to a reference (8‐step) calibration film taken at 5cm depth (95 cm SSD). (b) Curves fitted to percent differences between the calibrations at depth and the reference (8‐step) calibration. (c) Small (3×3 cm) and large (20×20 cm) field central axis film calibrations (XV and EDR) at 2mm depth (90 cm SSD) compared to the reference (8‐step) calibration at 5 cm depth (95 cm SSD).

The depth dependence of the 3×3 cm central axis small field calibration films taken at 90 SSD compared to the reference 8‐step calibration film are shown in Fig. 2(a). In addition to the depth dependence, the 8‐step calibration film has off‐axis exposure regions, resulting in an additional response difference of up to 1%. Differences between the depth‐dependent calibrations and the reference 8‐step calibration varied with depth and film type as shown in Fig. 2(b).

Differences in the film response for small (3×3 cm) and the large (20×20 cm) fields at 2 mm depth are shown in Fig. 2(c), shown relative to the 8‐step calibration response. The large field response is closer to the 8‐step calibration response than the small field response. This effect was attributed to the increase in scatter contributions with increasing depth or field size affecting the film response similarly.

**Figure 3:**
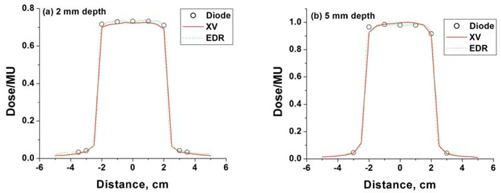
Film measurements (XV and EDR) compared to diode measurements at (a) 2 mm depth and (b) 5mm depth, for a 5×5 cm jaw‐field.

Good agreement between diode data and film (XV and EDR) for a 5×5 cm jaw‐field profile in the buildup region is shown in Fig. 3. Profile measurements for square fields agreed to within 3% for XV film and 2% for EDR film at buildup depths in non‐penumbral regions.

**Figure 4:**
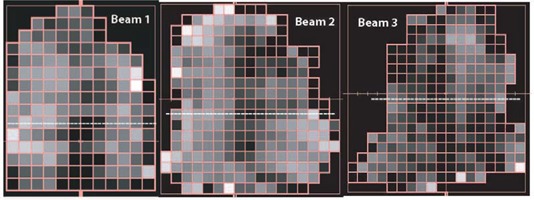
Intensity modulated fields. Diode measurements were performed at the center of each 1×1 cm beamlet along the dashed lines.

**Figure 5:**
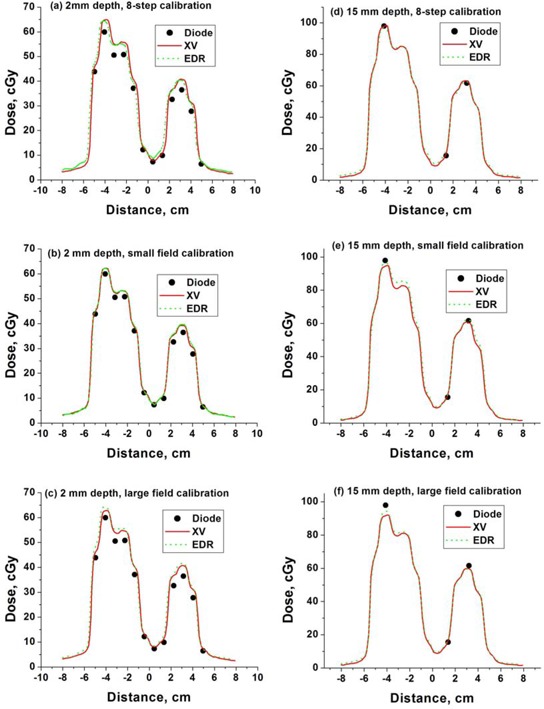
Film profiles (XV and EDR) obtained using (a) 8‐step, (b) small field and (c) large field calibration compared to diode measurements for IMRT beam #1 at 15 mm from central axis and depth of 2 mm (a‐c) and 15 mm (d‐f), respectively. EDR film response has been divided by a factor of 2.

The film response as a function of depth and field size was used to determine dose profiles, for example IMRT fields (as shown in Fig. 4). The IMRT fields ranged in monitor units from 85 to 206, and used up to 250 field segments. Film response was compared to diode measurements performed along the axes indicated. The film‐diode dose comparison was performed at 2 mm using the 8‐step calibration film as shown in Fig. 5(a), the large field response calibration as shown in Fig. 5(b), and the small field calibration as shown in Fig. 5(c). Measurements were also taken at 5 mm depth (not shown) and a sampling at 15 mm depth as shown in Figs. 5 (d‐f). XV and EDR film provided consistent results. The small field calibration technique was as good or better in all cases (profiles for beam 2 and 3 not shown). Calibration curve corrections relative to the 8‐step standard varied according to depth and dose level from −1% to 10%, with the largest corrections required for small field, shallow depth responses.

**Figure 6:**
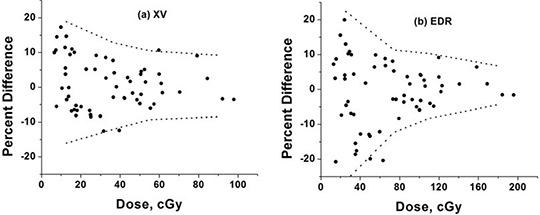
Residual errors between diode measurements and (a) XV and (b) EDR film measurements obtained using a small (3×3 cm central axis) field calibration for IMRT fields. The dashed lines represent approximate two standard deviations error.

The film‐diode measurement differences are shown in Fig. 6 for both film types, small field calibration and all beam profiles measured. Dotted lines mark the two standard deviation envelope. Similar errors were observed for both film types. Measurement error is smaller at larger doses and tends to have a modest bias toward over‐response at lower doses for both film types. The data show some systematic trends between the XV and EDR responses relative to the diode measurements, implying that the diode measurement error may have a non‐negligible role in the remaining discrepancies. Compared to XV, EDR response error was smaller at higher dose levels and larger at low dose levels.

**Figure 7:**
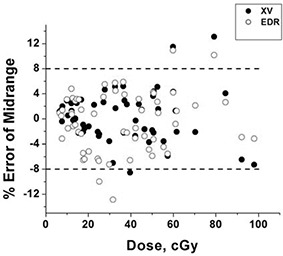
Residual errors between diode measurements and film measurements obtained using the small (3×3 cm central axis) field calibration relative to midrange dose (50 cGy for XV, 100 cGy for EDR exposures). Doses for EDR data were divided by two for display purposes. The dashed lines represent the approximate two standard deviation error of 8% of mid‐range dose.

Absolute measurement differences have less variation as a function of dose, as shown in Fig. 7, where the measured differences are presented as a percent of mid‐range dose (50 cGy for XV or 100 cGy for EDR). The two‐standard deviation level was ~8%.

## IV. DISCUSSION

Improved measurement of IMRT dose will improve our understanding of the tradeoffs implicitly carried out by the inverse‐planned optimization procedure. Inaccurate calculation can lead to more serious errors when using inverse planning techniques relative to forward planning techniques. Increased confidence in the calculation will lead to increased confidence in the planning process, improving the trade‐off decisions between target coverage and normal tissue sparing.

All measurement techniques have advantages and disadvantages. Film dosimetry was chosen for investigation because it offered high resolution and large area 2D distributions that could be summed to form a 3D distribution. Disadvantages include energy response difference compared to soft tissue and day‐to‐day variation sensitivity caused by film manufacturing differences and variations in processing environment. Both XV and EDR film were investigated to help judge the effects of energy dependence and the (film‐dependent) non‐linearity of dose response using a dynamic calibration technique. Others have used dynamic film calibration to account for changes in sensitivity with scatter conditions.^(10)^ ^,^ ^(20)^

The optimum film‐calibration field size was uncertain. While the intensity modulated field is delivered by a series of small fields, the in‐field scatter is characteristic of a much larger field size. Not surprisingly, for near‐surface depths, where the in‐field scatter contribution remains relatively low, the small field calibration was most appropriate. Use of improved knowledge of the particle and spectral components of the beam in the buildup regions may allow further reduction in film dose error.^(10)^ Use of a 3×3 cm field size for calibration was motivated by the desire to minimize the effects of scatter on film response, while maintaining a reasonable level of dosimetry accuracy (i.e., extent of uniform dose region) to minimize the impact of positional errors. Use of reference 3×3 cm fields at 5 cm depth served to relate results back to the original calibrations performed as a function of depth. Differences between the response at 5 cm depth and near‐surface depths were as high as ~10%. See Fig. 2(b).

EDR film is known to have less energy dependence, but also inferior signal response at lower doses compared to XV film. This trend is seen in the response difference error curves shown in Fig. 6. At the doses studied, an overall more reliable response was found from the XV film. However, this conclusion could be shifted in favor of EDR film at higher doses (e.g., additional repeat IMRT beam exposures per film). The diode‐film response differences attributed to film response error represent a nearly worst case scenario, since they encompass on‐ and off‐axis responses at multiple depths in addition to contributions from diode measurement error.

More recent film development has resulted in the improved usefulness of radiochromic films, such as EBT.^(21)^ ^,^ ^(22)^ While radiochromic film has the advantages of less energy dependence and ease of use (e.g., self‐developing), it has the significant disadvantage of being more noisy and prone to larger systematic error due to lower signal to noise ratios. Lynch et al.^(23)^ reported on EBT reponse dependence on scanning bed position, angular orientation and scanner temperature. Sankar et al.^(24)^ compared EDR2 to EBT film for IMRT field measurement and concluded that EBT film was more inconsistent for larger fields, but reliable for small field sizes. Others have verified EBT film's energy response independence.^(25)^ ^,^ ^(26)^

## V. CONCLUSIONS

Radiographic film dosimetry demonstrated sufficient accuracy (≤5% standard deviation) for the measurement of IMRT and other external beam dose in the near‐surface buildup region. A small field calibration was found to be most appropriate for dosimetry in near‐surface buildup regions of IMRT fields. XV film demonstrated superior performance relative to EDR film at typical beam doses. Film calibration corrections for near‐surface response of up to 10% were required relative to a reference at‐depth (5 cm) calibration. This technique can be used to acquire 2D dose distributions at near‐surface depths to evaluate (or commission) dose calculation algorithms for IMRT treatment planning.
